# Associations Between Race/Ethnicity, Language, and Enrollment on Cancer Research Studies

**DOI:** 10.1093/oncolo/oyac218

**Published:** 2022-11-02

**Authors:** Ogochukwu M Ezeoke, Gary Brooks, Michael A Postow, Shrujal Baxi, Soo Young Kim, Bharat Narang, Lisa C Diamond

**Affiliations:** Department of Pediatrics, Ann and Robert H. Lurie Children’s Hospital, Chicago, IL, USA; College of Medicine, State University of New York Upstate Medical University, Syracuse, NY, USA; Department of Medicine, Memorial Sloan Kettering Cancer Center, New York, NY, USA; College of Medicine, State University of New York Upstate Medical University, Syracuse, NY, USA; Department of Medicine, Memorial Sloan Kettering Cancer Center, New York, NY, USA; Weill Cornell Medical College, New York, NY, USA; Department of Medicine, Memorial Sloan Kettering Cancer Center, New York, NY, USA; Department of Medicine, Memorial Sloan Kettering Cancer Center, New York, NY, USA; Department of Medicine, Memorial Sloan Kettering Cancer Center, New York, NY, USA; Department of Medicine, Memorial Sloan Kettering Cancer Center, New York, NY, USA; Weill Cornell Medical College, New York, NY, USA; Department of Psychiatry and Behavioral Sciences, Memorial Sloan Kettering Cancer Center, New York, NY, USA

**Keywords:** healthcare disparities, language barriers, language equity, clinical trials, cancer research studies

## Abstract

**Purpose:**

The objective of this study was to determine whether differences in patients’ race/ethnicity, preferred language, and other factors were associated with patient enrollment in oncology research studies.

**Patients and Methods:**

We conducted a retrospective cross-sectional analysis of all adults (>18 and ≤90) seen at a large, metropolitan cancer center from 2005 to 2015, examining if enrollment to a research study, varied by race/ethnicity, preferred language, comorbidities, gender, and age.

**Results:**

A total of 233 604 patients were available for initial analysis. Of these, 93 278 (39.9%) were enrolled in a research protocol (therapeutic and non-therapeutic studies). Patients who self-reported their race/ethnicity as Native, Other, Unknown, or Refuse to Answer were less likely to be enrolled on a study. Patients with one or more comorbidities, and those whose preferred language was English, were more likely to be enrolled on a research study. A logistic regression model showed that, although Non-Hispanic Black patients were more likely to have one or more comorbidities and had a higher proportion of their subset selecting English as their preferred language, they were less likely to be enrolled on a study, than our largest population, Non-Hispanic/White patients.

**Conclusions:**

We identified differences in research study enrollment based on preferred language, and within race/ethnicity categories including Native-Populations, Other, Unknown or Refuse to Answer compared to Non-Hispanic/White patients. We also highlighted the lower odds of enrollment among Non-Hispanic/Black patients, in the setting of factors such as comorbidities and English language preference, which were otherwise found to be positive predictors of enrollment. Further investigation is needed to design targeted interventions to reduce disparities in oncology research study enrollment, with particular focus on language diversity.

## Teaser

This article reports on whether differences in patients’ race/ethnicity, preferred language, and other factors are associated with patient enrollment in oncology research studies.

**Implications for Practice**

Diverse enrollment on cancer research studies may be impeded by language barriers and the lack of adequate resources to support a multi-lingual trial population. During trial development, this is an area where particular focus may be dedicated to increase diversity in research studies. This study highlights the impact of language on enrollment on cancer research studies, discusses the implications of language diversity on increasing multi-ethnic study enrollment, and provides methods to support multi-ethnic/multi-lingual enrollment.

## Introduction

Non-White populations, including multi-ethnic, multi-lingual populations, are more likely to present with tumors with more aggressive biological behavior compared with Non-Hispanic/White patients at initial diagnosis.^1-8^ These factors contribute to an overall worse prognosis and decreased overall survival in these populations. Previous studies have examined the reasons for these disparities in presentation and outcome.^1,2^ These have included assessments of differences in disease manifestation, analyses of socioeconomic and education levels, and studies of genomic variations across race/ethnicity.^2,3^ Several investigations indicate a higher incidence among Non-White race/ethnicity groups for certain cancers.^1^ To understand the biology of the patients with cancer in the US, diverse cohorts are necessary; they provide clarity on any variations in cancer pathology by race/ethnicity or geographical ancestry, and promote patient centered therapy development.^9-11^

Literature examining overall participation in cancer research studies and investigating the role of race/ethnicity and language is sparse. Particularly regarding enrollment in studies for rare cancers and studies involving social influences of health. Research diversity improves the generalizability of cancer therapy, improves patient-reported outcomes, and allows the development of trials that support a variety of patient needs.^12-14^ Understanding the factors that may be associated with enrollment of study volunteers will inform the development of strategies to increase participation in cancer research studies. We aim to use the experience of a large urban cancer center to investigate how race/ethnicity, the presence of comorbidities, and preferred language may affect participation in oncology research studies.

## Methods

We performed a retrospective cross-sectional analysis of adult patients seen at an NCI-designated comprehensive cancer center between 2005 and 2015. We assessed variability in enrollment—defined as registration into any research study—based on race/ethnicity and preferred language. We collected data on patient gender and comorbidities, as these variables are part of inclusion/exclusion criteria for some research studies.

Race/ethnicity is self-reported by patients upon initial registration. Patients may select a specific race category, of the following: Asian, Black/African-American, Native-American or Native-Hawaiian, Other, Unknown, Refused to Answer or White. A free text response for Race was not an option during our study period. An ethnicity category also allows the following: Hispanic/Latinx, Not Hispanic, and Unknown. The institution in the last 10 years made the change to separate the race/ethnicity selections into 2 distinct options. In order to assess data similarly prior to and following this change, we combined both variables. If participants indicated that they were Hispanic or Latinx, this was coded as their race/ethnicity variable, otherwise only the selected Race category was recorded. Eight mutually exclusive race/ethnicity categories were established: Non-Hispanic/White (NHW), Non-Hispanic/Black (NHB), Asian (AN), Hispanic/Latinx (HL), Native Populations (NP), Other (OT), Refused to Answer (RF), and Unknown (UKN). Preferred language is self-selected by patients upon registration out of 94 options. Preferred language was dichotomized into English or Non-English. Protocol exclusion by preferred language was defined by requirements for native English or equivalent fluency. Patient gender is self-selected at time of hospital registration, out of gender options: male, female, and the option to leave blank. To avoid theorizing upon missing data on gender, we did not include patients for whom these data were missing, in final analyses.

Comorbidities were identified using ICD-10 coding in patient electronic medical records. We included diabetes, coronary artery disease and hypertension, based on their high prevalence in the adult US population.^15^ We identified 2155 research protocols available over the time span of our study, including study types of Therapeutic (*n* = 1514), Diagnostic (*n* = 265), Clinical Genetic (*n* = 23), Epidemiologic (*n* = 58), Prevention (*n* = 19), Psychosocial (*n* = 126), Quality of Life (*n* = 106), and Specimen Banking (*n* = 44). We reviewed available eligibility criteria for protocols which had electronic eligibility documentation within the hospital record (*n* = 1647), to evaluate for exclusion based on our 3 selected comorbidities. We coded exclusion criteria in studies using criteria listed on www.clinicaltrials.gov by disease system (Cardiovascular, Endocrine, Hepatorenal, etc.), and focused on Cardiovascular and Endocrine to capture our comorbidities of interest (diabetes, coronary artery disease, and hypertension). The presence of comorbidities was determined as positive ICD-10 code for any of the 3 selected comorbidities.

We assessed for variation in enrollment, while accounting for study exclusion criteria related to comorbidities and Non-English preference. Using unique anonymizing identifiers, patients were stratified into enrolled vs. not enrolled in at least one research study. Research studies included the following protocol types: clinical genetics, diagnostic, epidemiologic, pediatric, preventive, psychosocial, quality of life, specimen banking, and therapeutic/clinical trials. We excluded from our analysis all patients <18 and ≥90 years of age, as well as patients with no value listed for race/ethnicity, language or gender.

Descriptive analyses used frequencies (%) and means (SD) as appropriate to the data. Bivariate associations of enrollment status by variables of interest used chi-square analyses. We assessed, with NHW—the largest cohort—as the reference group, the odds ratio of enrollment on a research study by race/ethnicity, language, gender, and presence of comorbidities. We performed all analyses using SAS, version 9.4. These analyses were approved by the cancer center’s IRB.

## Results

A total of 233 604 patients were available for initial review. This included all patients seen at the cancer center between 2005-2015 with a cancer diagnosis. Of these, 93 278 (39.9%) were enrolled in at least one study. Table 1 shows research study enrollment by patient demographics and comorbidities. The race/ethnicity breakdown of the total initial population was: NHW: 74.6%, NHB: 5.5%, AN: 4.6%, HL: 4.2%, NP: 0.1%, OT: 0.5%, RF: 3.5%, and UKN: 3.6%. When assessing for variability in enrollment proportions by race/ethnicity alone, differences in the NHW (43.2%), NHB (44.0), AN (42.9), and HL (42.3), groups were small, although statistically significant (*P*-value <.0001). When including the percent enrolled within the categories of NP (27.2%), OT (38.1%), RF (33.2%), and UNK (3.4%), we see notable absolute value differences (*P*-value <.0001). Populations of patients selecting UNK (8445; 3.6%) or RF (8174; 3.5%), represented the fifth and sixth largest cohorts seen at the cancer center, respectively, with the NHW category being the largest (174 284; 74.6%).

**Table 1.** Research study enrollment by race/ethnicity, gender, comorbidity status, and English language preference.

	All subjects		Enrolled		Not enrolled		*P*-value^*^^/^^**^
	*N*	Percent of total	*N*	Group percent	*N*	Group percent	
Race/ethnicity categories							
Non-Hispanic/White	174 286	74.6	75 304	43.2	98 982	56.8	
Non-Hispanic/Black	12 963	5.5	5702	44.0	7261	56.0	
Asian	10 805	4.6	4639	42.9	6166	57.1	
Hispanic/Latinx	9727	4.2	4116	42.3	5611	57.7	
Native populations	162	0.1	44	27.2	118	72.8	<.0001*
Other	1119	0.5	426	38.1	693	61.9	
Refused to answer	8174	3.5	2714	33.2	5460	66.8	
Unknown	8445	3.6	290	3.4	8155	96.6	
Missing^+^	7923	3.4	43	0.5	7880	99.5	
Gender							
Female	124 345	53.2	49 307	39.7	75 038	60.3	.0036*
Male	109 254	46.8	43 969	40.2	65 285	59.8	
Unknown (missing^+^)	5	0.0	2	40.0	3	60.0	
Comorbidities							
1 or more	100 737	43.1	49 398	49.0	51 339	51.0	
None	132 867	56.9	43 880	33.0	88 987	67.0	<.0001*
English preferred							
Yes	185 587	79.4	81 984	44.2	103 603	55.8	
No	11 407	4.9	3544	31.1	7863	68.9	<.0001*
Missing^+^	36 610	15.7	7750	21.2	28 860	78.8	
Mean age (years) (±SD)	57.9(±16.0)	n/a	57.7(±13.7)	n/a	58.9(±14.6)	n/a	<.0001**
Total	233 604	100.0	93 278	39.9	140 326	60.1	

*P*-values are from Chi-square tests of independence.

*P*-value from Mann-Whitney U test.

Patients with missing data were not included in final analysis

A larger proportion of patients overall were of female identifying (53.2% vs 46.8%, *P*-value .0036). Differences in enrollment by gender were small, with female patients less likely to be enrolled compared to male patients (39.7% vs 40.2%: *P*-value <.0036). Although these results show statistical significance, it is difficult to determine the clinical significance of this difference. A higher proportion of patients with ≥1 comorbidities was enrolled in a study (49.0%) compared to patients with no comorbidities (33.0%) (*P*-value <.0001). A higher proportion, of those with English language preference, was enrolled in a study (44.2%) compared with those whose language preference was not English (31.1%) (*P*-value <.0001).

Table 2 is a breakdown of the total initial population by race/ethnicity, by the presence of comorbidities, selection of female gender, and preferred language. The subset of patients selecting NHB had a larger proportion with ≥1 comorbidity compared with all other race/ethnicity categories (58.4% vs NHW: 45.6%, AN: 43.2%, HL: 48.0%, NP: 50.6%, OT: 41.0%, RF: 38.8%, and UNK: 5.9%; *P*-value <.0001). The NHB population was most likely to report English as their preferred language, while patients in the UNK category, represented the smallest proportion of patients to do so (NHB: 89.3% vs NHW: 85.1%, AN: 73.1%, HL: 66.0%, NP: 85.2%, OT: 74.1%, RF: 84.8%, and UNK: 20.5%; *P*-value <.0001).

**Table 2.** Total population: comorbidities, gender, and English language preference by race/ethnicity categories.

	Has 1 or more comorbidities			Female gender		English language preferred	
	Total population	Total^a^	% of total population	Total^b^	% of total population	Total^c^	% of total population
Race/ethnicity categories							
Non-Hispanic/White	174 286	79 546	(45.6)	91 281	(52.4)	148 253	(85.1)
Non-Hispanic/Black	12 963	7,569	(58.4)	7,873	(60.7)	11 573	(89.3)
Asian	10 805	4666	(43.2)	6393	(59.2)	7899	(73.1)
Hispanic/Latinx	9727	4671	(48.0)	5682	(58.4)	6416	(66.0)
Native populations	162	82	(50.6)	89	(54.9)	138	(85.2)
Other	1119	459	(41.0)	571	(51.0)	829	(74.1)
Refused to answer	8174	3175	(38.8)	4039	(49.4)	6934	(84.8)
Unknown	8445	496	(5.9)	4241	(50.2)	1729	(20.5)
Total	233 604	100 737	(43.1)	124 345	(53.2)	185 587	(79.4)

All values are *N* (%).

All *P*-values <.0001 (Chi-square test of independence).

Total with 1 or more comorbidities.

Total who identified as female at hospital registration.

Total who selected English as the clinical language preference.

Table 3 examines patients without comorbidities, highlighting the variation by race/ethnicity between those selecting English, and those selecting any of the 94 other languages. Of patients without comorbidities, only 5.9% of those who selected both UNK and English, are enrolled. This is the lowest overall proportion when compared with the percent enrolled within all other race/ethnicity categories (5.9% vs NHW: 39.9%, NHB: 38.9%, AN: 42.4%, HL: 43.2%, NP: 24.2%, OT: 38.1%, and RF: 30.5%; *P*-value <.0001). Among those who preferred to communicate in a language other than English, the NP and AN categories were the smallest proportion of those enrolled in a study (NP: 0% and AN: 11.7% vs NHW: 26.0%, NHB: 13.6%, HL: 13.6%, OT: 28.2%, RF: 18.9%, UNK: 12.9%; *P*-value <.0001).

**Table 3.** Total population: subset of patients with no comorbidities by English language preference.

	English language preference			Non-English language preference		
	Total^*^	Total^*^ enrolled	% Total^*^ enrolled	Total^^^	Total^^^ enrolled	% Total^^^ enrolled
Race/ethnicity categories						
Non-Hispanic/White	80 192	31 986	(39.9)	1529	398	(26.0)
Non-Hispanic/Black	4712	1831	(38.9)	44	6	(13.6)
Asian	4639	1967	(42.4)	929	282	(11.7)
Hispanic/Latinx	3577	1545	(43.2)	899	268	(13.6)
Native populations	66	16	(24.2)	4	0	(0.0)
Other	478	182	(38.1)	71	20	(28.2)
Refused to answer	4264	1299	(30.5)	628	119	(18.9)
Unknown	1403	84	(5.9)	31	4	(12.9)
Totals	101 097	38 926	(38.5)	4230	1098	(25.9)

All values are *N* (%).

All *P*-values <.0001 (Chi-square test of independence).

No comorbidities with English language preference.

No comorbidities with Non-English language preference.

Of the 1647 studies with available eligibility criteria, 1451 (88.1%) excluded patients based on cardiovascular or endocrine disease, which include our selected comorbidities. Of these studies, 218 (13.2%) excluded patients based on non-English preferred language. Exclusion by preferred language was defined as English fluency insufficient to the point of requiring study translation.

**Figure 1.**
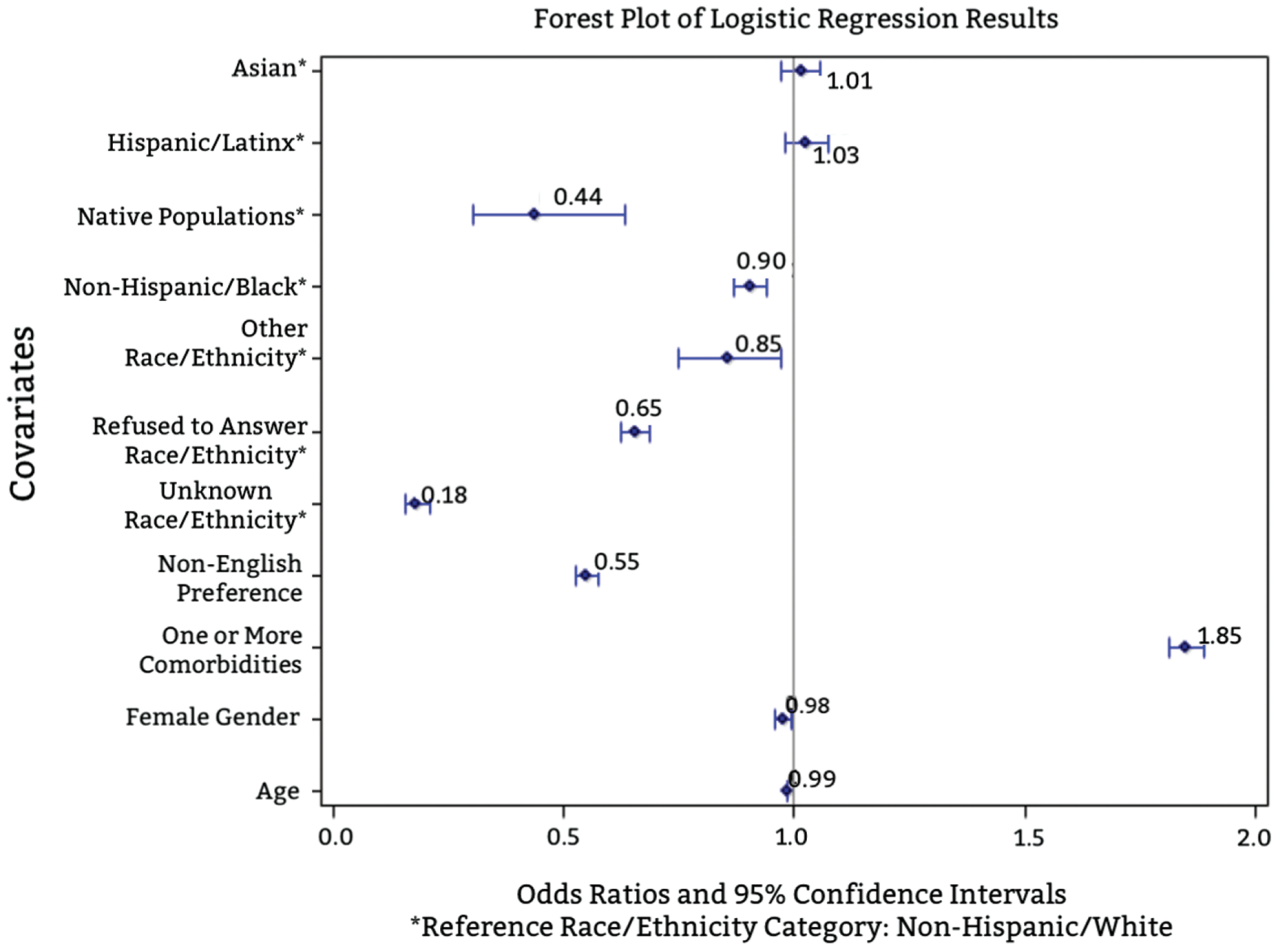
Logistic regression for research study enrollment.

Figure 1 shows logistic regression assessing multifactorial odds ratio of enrollment. There were reduced odds of enrollment in the NHB (OR: 0.91, 95% CI: 0.87, 0.94), RF (OR: 0.65, 95% CI: 0.62, 0.69), NP (OR: 0.44, 95% CI: 0.31, 0.63), UNK (OR: 0.18, 95% CI: 0.16, 0.21), and OT (OR: 0.85, 95% CI: 0.75, 0.97) categories. Patients who preferred non-English languages had reduced odds of enrollment in a research study (OR: 0.55, 95% CI: 0.53, 0.58). Logistic regression showed slightly reduced odds for female identifying patients being enrolled (OR = 0.98, 95% CI: 0.96, 0.99). Patients with one or more comorbidities had increased odds of enrollment (OR: 1.85, 95% CI: 1.81, 1.89). For each 1-year increase in age, the odds of being enrolled in a study decreases by 0.015.

## Discussion

Our data show that statistically significant differences exist in enrollment by race/ethnicity, on a wide range of research studies. The practical significance of differences seen between NHW, NHB, HL, and AN is difficult to determine, with the percent enrollment, per group, all just above 40%. On the contrary, there is a potentially actionable difference in the enrollment of those in the NP, OT, RF, and UNK groups, with a range from 3.4% in UNK to 38.1% in OT. Although all results show statistical significance, with *P*-values <.05, we must apply nuance and recognize that further investigation is needed to scrutinize and interpret small differences seen between NHW, NHB, HL, and AN groups. The sharper disparity seen both between NP, OT, RF, and UNK, and as compared to NHW, NHB, HL, and AN, warrants examination for action, particularly as relates to patients selecting race/ethnicity as UNK. This group had the lowest proportion selecting English as the preferred language for clinical care and the lowest proportion with ICD-10 codes for any of our selected comorbidities.

We see that the presence of comorbidities appears to be a positive factor toward enrollment in a research study. The NHB population had higher incidences of one or more comorbidities, however they still appear to have lower odds of being enrolled overall on a study, as seen in our logistic regression. Additionally, although the selection of English as a preferred language appears to be associated with an increased odds of enrollment on a research protocol, this, once more, does not hold true within our NHB population, despite having a high percentage of patients with English as their preferred language. While there is an association between language selection and enrollment on a research study, there remains nuance in how these results can be interpreted across different populations.

As we uncover the role of language on enrollment, we recognize that studies have highlighted difficulties in research informed consent, when language barriers exist.^16-18^ While we observed exclusion in studies based on preferred language, this may be more clearly explained as exclusion based on challenges in translating study materials into non-English languages and gaps in resources to devote to professional interpreter use in study visits. The ability to translate into plain English the implications and requirements of a research study is one which oncologists practice daily. However, the insertion of a third party into the discussion, such as an interpreter, the lack of an established relationship with a physician, and the absence of trust in the healthcare system could all limit successful communication.^16,19^ Regarding the protocol documents, including consent and any additional patient forms associated with the study, there is a dependence on the industry sponsor or NIH-fund to facilitate the translation to any potential written language for use by the patient. However, even with translation of documents, the consent process requires the additional layer of culturally informed clinical discussions in the appropriate language.

It appears that language barriers may not be the cause of lower cancer research enrollment in our NHB population. The unfortunate historical context of the institution of medicine and the NHB community includes numerous actions which have significantly impeded trust. These actions include prominent examples of large-scale NHB research enrollment include the Tuskegee Syphilis Study and the Baltimore Lead Paint Study.^20-22^ The second lowest proportion for enrollment is seen in NP, who, like the NHB population, has a high ratio of the cohort selecting English as their primary clinical communication language, and has a high incidence of one or more comorbidities. Here, once more we evaluate these results by looking at historical context. In reviewing the literature, Findling et al show reports of discrimination by Native Americans, and potential avoidance of healthcare interaction—which would be required for participation in a research study—for fear of discrimination. This may be a consequence of historical injustices—as discussed by Walters and Simoni—that has tainted the trust between Native Americans and the healthcare system.^23-25^

Medicine will require rebuilding of trust, educating clinicians about the context with which NP, NHB and limited English proficient (LEP) patients arrive at the hospital. Through this exercise we may be able to better address the communication barriers that exist within cancer trial informed consent. The cancer center is working to increase research enrollment for previously underrepresented participants, including LEP patients, through a core facility which advises principal investigators on community outreach and engagement.

It is worth noting that patients with the presence of at least one comorbidity represented a larger proportion of total enrolled patients. This is perhaps secondary to an established relationship with a healthcare provider, and potentially positive experiences. In examining the literature, it is clear that having a relationship within oncology or clinical care plays a significant role in commitment of a patient to joining a clinical trial.^26^ Enrollment in research studies is often guided by the presence of, and ability to manage comorbidities in patients, particularly as there is a perceived increase in toxicity in these trial participants.^27,28^ These stringent requirements do not necessarily extend to the enrollment of patients on non-therapeutic research protocols. There were 2155 research protocols available over the time span of our study, of which therapeutic (*n* = 1514) and diagnostic (*n* = 265) studies made up 82.6%. Within this selection, only 76.4% (*n* = 1647) had available inclusion and exclusion criteria in an accessible electronic format. Additional included study types were Clinical Genetic (*n* = 23), Epidemiologic (*n* = 58), Prevention (*n* = 19), Psychosocial (*n* = 126), Quality of Life (*n* = 106), and Specimen Banking (*n* = 44). Within individual trials, we must acknowledge that there are likely specific exclusion criteria which our broad based evaluation would not capture, particularly regarding clinical exclusions or comorbidities. Additionally, the identification of race/ethnicity differences in enrollment within therapeutic versus non-therapeutic trials would have added significant value to our study; it would have allowed us to evaluate the role of therapeutic benefit in patient enrollment decisions. As many patients were enrolled in more than one study over our assessment period, we focused on enrollment to any study rather than enrollment by trial type, to avoid duplicate counting. The increased enrollment among patients with comorbidities was not observed in the NP or NHB subgroups. There are a variety of possible reasons for this finding, including mistrust in medical systems as noted above. This mistrust among NP and NHB patients, with comorbidities, may be exacerbated by increased exposure to the medical system, where reports of discrimination are seen at greater proportions when compared with NHW populations.^23-25,29^ We must also consider that clinicians may be reluctant to refer patients to clinical trials, due to reasons including lack of resources to address minoritized populations such as NP and NHB, with culturally appropriate recruitment and retention strategies.^30,31^ The cancer center has responded to these known biases through its Office of Health Equity. Specific strategies currently being used include a detailed evaluation of disparities in clinical trial enrollment for any open studies at the center, an assessment of possible barriers to diverse enrollment for studies in the development phase and the identification of trial enrollment benchmarks to strive toward.

Our study underlines stark differences in enrollment in the categories of NP (27.2%), OT (38.1%), RF (33.2%), and UNK (3.4%) compared with other categories and shows that those who selected UNK were least likely to have selected English as their preferred language. The reporting of race/ethnicity is part of the registration process of new patients to the cancer center, to allow the evaluation of equitable care throughout the course of a patient’s treatment.^32^ It is a voluntary segment of the registration process and our results compel us to consider 2 factors: the role of race/ethnicity in perceived medical care and the understanding of US-based social constructions of race/ethnicity. The perception that selecting one race/ethnicity category may impact the quality of care being delivered is not unique amongst, for instance, the NHB community.^33^ A patient may perhaps perceive that selecting UNK may be preferable when hoping for the excellent care that is offered at a large institution such as the study cancer center. The second consideration we have is the understanding of these categories within the international, ­non-USpopulation. The social construct of race remains a concept that does not fully capture the geographical ancestry of patients across the globe who may present to a large center for cancer care. Patients may select UNK if they do not find the race/ethnicity categories applicable to their own racial/ethnic identity. The selection of UNK, combined with any additional language barriers, may also create a perception among clinicians that a language barrier and informed consent might be challenging.^34^ The result, regardless, is a decrease in enrollment of patients who do not fit within the demarcations of historical race/ethnicity categories.

Previous research that has highlighted disparities in enrollment in cancer research studies, has focused on specific cancer diagnoses.^3,35-39^ Our analysis is unique in that we evaluate all available studies, including tumor banking studies, which may not, for instance, require the same time or trust commitment as might therapeutic trials.^40^ Cancer research studies allow us to tackle a variety of questions related to patient care, including among many, quality of life, treatment response, and cancer genomics. Given the broad nature of our study, we are able to address the question of enrollment influences across the spectrum of research studies, with and without the consideration of direct therapeutic benefit to the enrolled patient. Within the selection of protocols which were available during our study period, the majority were interventional in nature including therapeutic (*n* = 1514, 70.3%) and diagnostic clinical trials (*n* = 265, 12.3%); however, several other study types were available. Our approach also provides a reflection on additional broad variables which may impact enrollment, including pregnancy as an exclusion criteria for many studies,^41^ and the availability of studies for disease affecting one gender over the other. For instance, an analysis of NIH-funded studies by Mirin et. al.,^42^ shows increased overall funding for studies affecting men whereas there was on average underfunding for studies targeted at diseases primarily affecting women.^41^ These factors could certainly impact the enrollment numbers which we found, particularly with respect to gender where women (39.7%) were enrolled at a lower proportion compared to men (40.2%). We are able to take a broad approach to ameliorating the impact of perceived language barriers, as well as mistrust of the medical community. Critically, enrollment goals for every study must be clarified during trial development; ensuring a diverse study population requires stating unambiguously the need for diverse recruitment. Meaningful goals for multi-ethnic enrollment should at least reflect the population demographics. To facilitate this effort, the inclusion of community leaders and clinicians as collaborative investigators is necessary. Although there should be ongoing diversification of the primary site clinician workforce, seeking out local practitioners from diverse, multi-lingual communities may ease discussions around research enrollment with patients who may be initially hesitant to be involved. Studies have repeatedly highlighted the improvement in ­physician-patient communication, where race concordance exists,^43^ and this data should be used to the advantage of research study developers. Finally in reviewing the criteria for enrollment on research studies, ensuring diversity amongst reviewers, may allow nuanced conversations around who may be unintentionally excluded, via criteria which may not significantly impact the success of the study.

## Limitations

Although this is a large metropolitan hospital which serves 23 counties of an estimated 18 million people, with 53% identifying as Non-White, a community health needs assessment conducted by the cancer center, found that in 2015, <20% of the cancer center’s patient population was diverse. The assessment reported perceptions, by patients, of exclusivity.^44,45^ We acknowledge the difficulty in capturing a diverse cohort of patients with this perception likely influencing patient presentation to the hospital.

An additional limitation has been our ability to fully capture the role of comorbidities; as clinician assessment of these comorbidities was often the determining factor for participation on a trial, this data would have been beneficial for analysis. Comorbidity data in the electronic medical record are often incomplete^46^ and the center EMR was unfortunately not an exception, with sometimes inconsistent inputs of each clinical diagnosis, recorded as ICD-10 codes. Regarding which diagnoses to include in our study analysis, our selection was thus based on most common US adult chronic health conditions, which were also most consistently recorded in the medical record. We recognize the limitation of this list, not including a comprehensive selection of all comorbidities which may impact patient enrollment. We also acknowledge the limitation within the medical record surrounding timing of comorbidity diagnosis; as these records are timed based on clinician input into the EMR, they do not always correspond to initial diagnosis, and do not consistently reflect ­pre-enrollment comorbidities.

We also recognize that the availability of clinical trials varies by cancer type and that there are variations in cancer incidence by patient geographical ancestry. Further analysis, stratifying disease type by our race/ethnicity categories would have clarified these variations. We also acknowledge the limitation of the race/ethnicity categories, as they have been changed in the last decade. The ability to assess race/ethnicity variation in healthcare delivery, and thus identify opportunities for improving equitable care is limited when patients may not feel comfortable selecting a racial category. Equity research is improved when patients are provided with the options to most accurately describe their identity. Although we have found decreased enrollment amongst patients in the Native-Populations, Other, Unknown or Refuse to Answer categories, these findings would be more specifically actionable with the inclusion of more personalized patient data. We are able to infer potential barriers, based on the literature on clinical trial enrollment^47-49^, but it is a challenge to investigate more precisely into variations by race/ethnicity without improvements in how race/ethnicity data is captured. We also acknowledge the limitation of a binary-favoring gender system that does not reflect a diverse gender identity. The cancer center has responded to these known biases through its Office of Health Equity with a re-evaluation of the process for demographic data capture, including how race/ethnicity and gender information are collected.

## Conclusions

Our study highlights potential areas for improvement in the approach to increasing diversity in cancer research enrollment; while we continue to work toward increasing racial/ethnic diversity in cancer research enrollment to reflect the patient population who would benefit from research studies, we must realize that language barriers may also be impeding our efforts. We must also acknowledge the potential positive impact of culturally considered care on recruitment to cancer research studies should not be ignored. Enrollment in cancer research studies by a diverse cohort allows adequate interpretation of results that is beneficial to a multi-national patient population and provides insight into potential differences in pathology, as well as drug pharmacokinetics and pharmacodynamics across geographical ancestry.^2,50^ Language barriers create limited research study populations, in addition to known disparities in healthcare.

## Data Availability

The data underlying this article will be shared on reasonable request to the corresponding author.
